# Hemodynamic Effects of A Simplified Venturi Conduit for Fontan Circulation: A Pilot, In Silico Analysis

**DOI:** 10.1038/s41598-020-57634-2

**Published:** 2020-01-21

**Authors:** Fang Zhu, Guocheng Shi, Chen Wen, Qian Zhang, Qihua Fu, Jinlong Liu, Zhongqun Zhu, Huiwen Chen

**Affiliations:** 1 Department of Cardiothoracic Surgery, Shanghai Children’s Medical Center, Shanghai Jiao Tong University School of Medicine, Shanghai, China; 2 Department of Laboratory Medicine, Shanghai Children’s Medical Center, Shanghai Jiao Tong University School of Medicine, Shanghai, China

**Keywords:** Cardiac device therapy, Congenital heart defects, Fluid dynamics

## Abstract

Objectives: To study the effects of a self-powered Fontan circulation in both idealized Fontan models and patient-specific models. Methods: In silico, a conduit with a nozzle was introduced from ascending aorta into the anastomosis of superior vena cava and pulmonary artery. Computational fluid dynamics (CFD) simulation was applied to calculate the fluid fields of models. Three 3-dimentional idealized models with different offsets were reconstructed by computer-aided design to evaluate the effects of the self-powered conduit. Furthermore, to validate the effects in patient-specific models, the conduit was introduced to three reconstructed models with different offsets. Results: The pressures at superior venae cavae and inferior venae cavae were decreased in both idealized models (0.4 mmHg) and patient-specific models (0.7 mmHg). In idealized models, the flows to left lungs were decreased (70%) by the jets from the conduits. However, in patient-specific models, the reductions of blood to the left lungs were relatively limited (30%) comparing to idealized models. Conclusions: CFD simulation was applied to analyze the effectiveness of the Fontan self-powered conduit. This self-powered conduit may help to decrease the venae cavae pressures and increase the flow to pulmonary arteries.

## Introduction

The Fontan procedure has evolved over the decades since its first introduction in 1971^1^. Total cavopulmonary connection (TCPC) has been the most widely adopted Fontan procedure, in which an intra-atrial lateral tunnel or an extracardiac conduit is constructed to reroute blood from the inferior vena cava (IVC) to the pulmonary arteries^2^. Despite the accepted early survival rate following the Fontan procedure, bypass of the subpulmonary ventricle may inevitably result in long-term complications including protein-losing enteropathy^3^ and Fontan-associated liver disease^4,5^ in some survivors, which are partly due to increased systemic venous pressure^6^.

Multiple groups have attempted to provide an adequate preload to optimize the Fontan circulation using biological (contractile properties of the skeletal muscle^7^, tissue engineering/regenerative medicine devices^8^) or mechanical assist devices (i.e. the Von Karman impeller pump^9^, axial flow devices^10^, and the catheter-based device^11^). Moreover, though several reports were previously published, in which patients had focused on the use of the Berlin Heart^12^, the assist devices were large in size and complex in structure. This reflects the great barrier to translating these experimental studies into clinical practice. Thus, pursuit of a desirable, sustainable, and clinically feasible solution to improve late prognosis in the Fontan population is urgently required.

Several studies performed computational fluid dynamics (CFD) and aimed to achieve an ideal cavopulmonary connection^13,14^. A Venturi jet was designed to assist bidirectional Glenn in a previous study, but the increases in pulmonary flow were generally accompanied by increases in superior vena cava (SVC) pressure^15,16^. Ni and colleagues designed a self-powered Fontan circulation^17^. However, they did not assess the influence of offsets and different insert positions of the conduit and did not apply the conduit to patient-specific models. Thus, its clinical application requires further investigation. In the present study, we report a feasible and simplified Fontan self-powered conduit based on the Venturi effect and evaluate its role in reducing vena cava pressures and increasing pulmonary flow.

## Materials and Methods

### Ethical approval and informed consent

The Ethics Committee of the Shanghai Children’s Medical Center reviewed and approved this study. All procedures performed in studies involving human participants were in accordance with the ethical standards of the institutional and/or national research committee and with the 1964 Helsinki declaration and its later amendments or comparable ethical standards. Informed consent was obtained from the patients’ parents (patients were under 18 years old).

### Assist conduit

**Figure 1:**
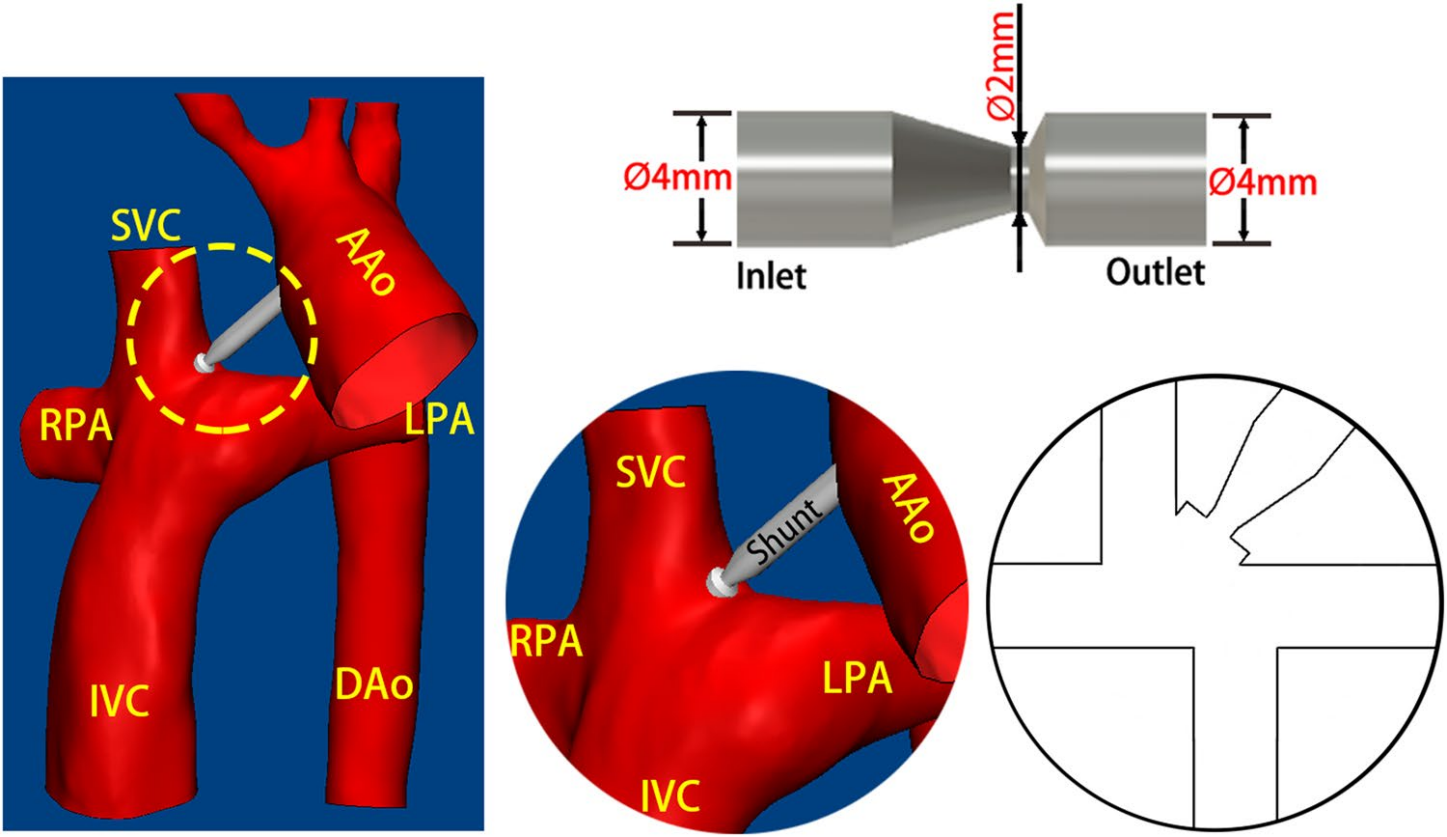
The schematic diagram of the assist conduit.

According to previous studies^15,17^, a conduit with a 2-mm nozzle has clinical usability. Therefore, our conduit consists of 3 sections: inflow, constricted, and outflow sections, whose diameters were 4 mm, 2 mm, and 4 mm, respectively (Fig. 1).

### Baseline model

The extracardiac Fontan post-operative models without assist conduit were set as baseline. Then the assist conduit would be added to the baseline models in silico to build experimental group.

### Idealized fontan model

**Figure 2:**
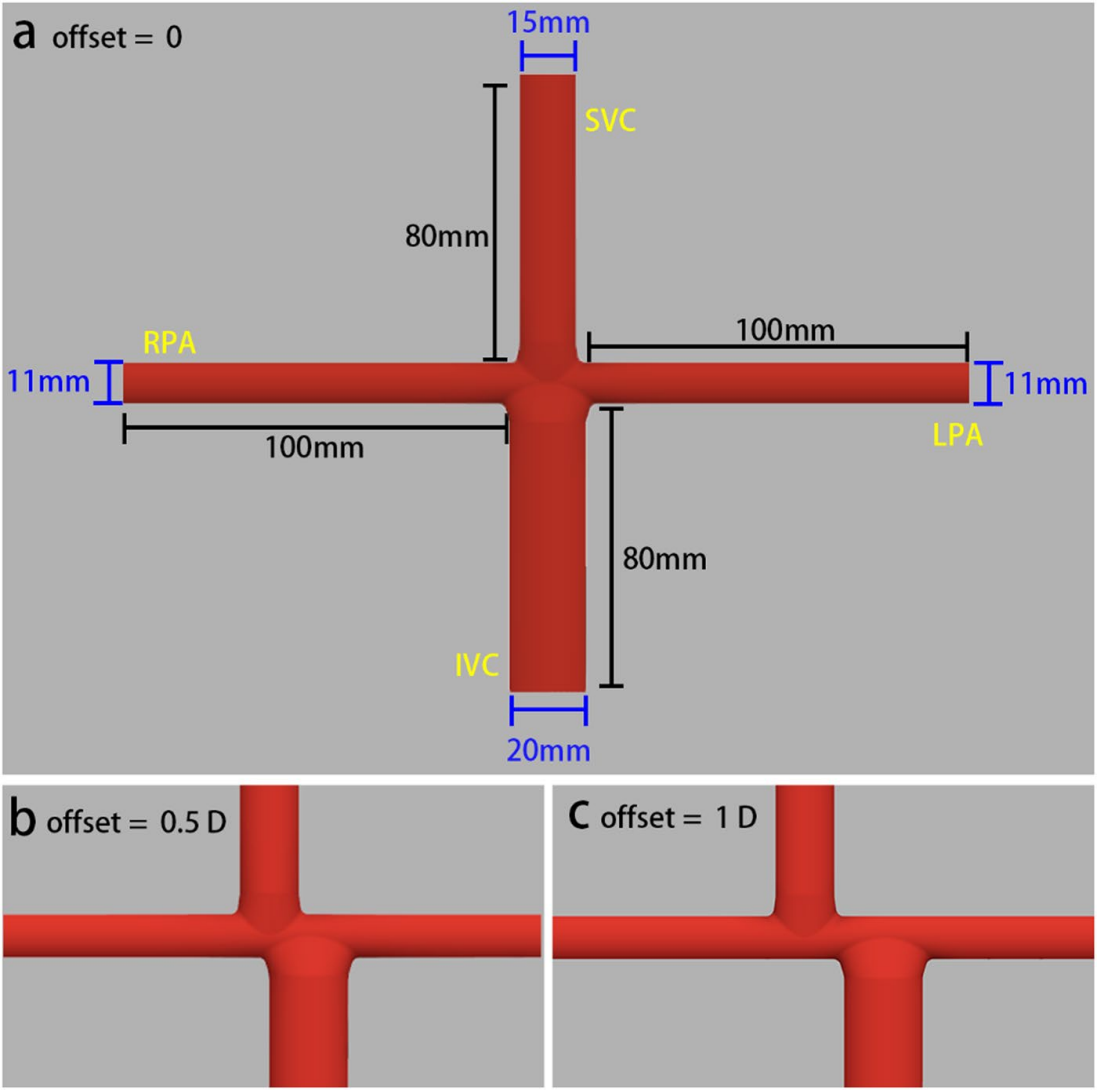
Idealized Fontan Models with different offsets. *SVC*, superior vane cava; *IVC*, inferior vena cava; *LPA*, left pulmonary artery; *RPA*, right pulmonary artery; *D*, diameter of inferior vena cava.

To construct an idealized Fontan model, 28 patients who underwent the extracardiac Fontan procedure during the second half of 2017 were selected (Table 1). At least one of cardiac computed tomography (CT), magnetic resonance imaging (MRI), and catheterization were performed before their discharge. The idealized model was constructed using Autodesk Fusion 360 (Autodesk, San Rafael, CA, US) with average dimensions of the patients (Fig. 2). The diameters of the left pulmonary artery (LPA), right pulmonary artery (RPA), and SVC were 11 mm, 11 mm, and 15 mm, respectively. The diameter of the IVC connection was set to 20 mm^18^. The different offsets between the SVCs and IVCs were set. The weight and height of all patients were measured to calculate their body surface area.

**Table 1:** Patient Characteristics. Continuous variables are presented as mean ± standard deviation. BSA, body surface area.

Characteristic	Value
Age, y	4.64 ± 2.35
Male/female, n	16/12
Heart rate at rest, beats per minute	106 ± 14.57
Mean arterial pressure, mmHg	65 ± 8
Height, cm	103.71 ± 14.57
Weight, kg	17.16 ± 5.82
BSA*, m^2^	0.70 ± 0.16

### Insert position

According to our clinical experience, the longer the conduit is, the easier it is to bend. Thus, the assist conduit was assumed to introduce the blood from ascending aorta to reduce the risk of bending. Considering the anatomies after the Fontan procedure, we select three insert positions for each Fontan model. The rules for inserting the conduit were formulated as follows:

1. The point of intersection of the centerlines of the RPA and SVC was the origin, which was denoted by the letter O. The direction along the LPA was the x-axis, the direction along the SVC was the y-axis, and the direction toward the front was the z-axis (Fig. 3a).

**Figure 3:**
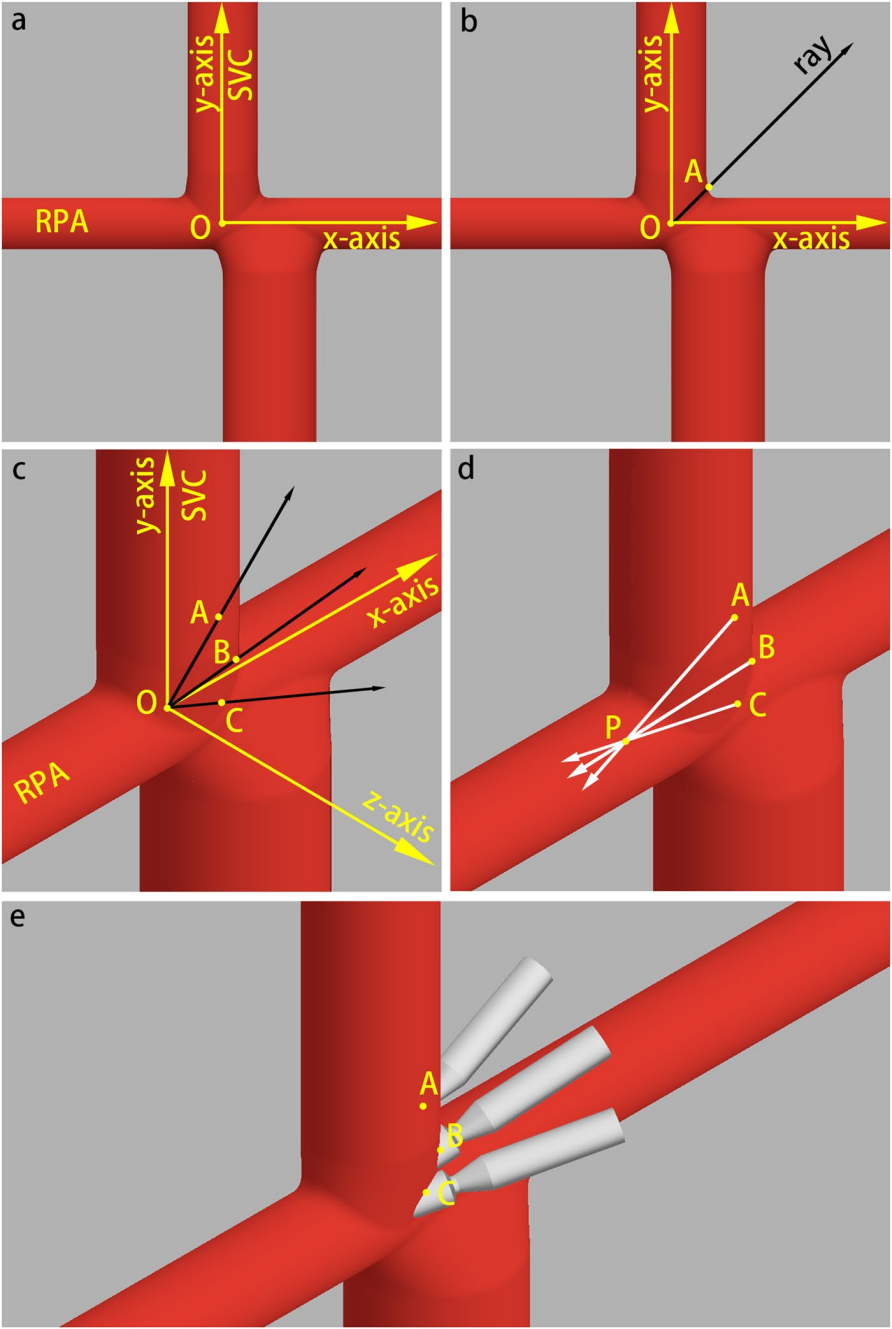
Insert positions of the idealized models. (**a**,**b**) Are the front views of the model. (**c**–**e**) Are the front-right-top view of the model. Point O is the intersection of the centerlines of the RPA and SVC. Point A, B and C are insert positions. Point P is the midpoint of the entrance of RPA. (**c**) Shows the relationship of point A, B, C and O (∠*AOB* = ∠*BOC* = 30°). (**d**) Shows the insert directions: three insert directions point toward point P. (**e)** Shows the difference of positions and directions of the three insert points.
2. In the x-y plane, a ray was graphed from the origin having a 45° angle with the x- and y-axes. The intersection of the ray and the wall of the Fontan pathway was set as point A (Fig. 3b).
3. The ray was rotated 30° toward the positive z-axis. At this time, the intersection of the ray and the wall was set as point B, and then rotated again to create point C (Fig. 3c).
4. After setting three insert positions, the self-powered conduits were inserted from each point (A to C) toward point P (midpoint of the entrance of RPA) (Fig. 3d,e). In an idealized situation, 3 baseline models and 9 models with self-powered conduits were constructed.

### Patient-specific models

In this computer simulation analysis, we collected the clinical data of three patients who had underwent the extracardiac Fontan procedure at the age of 5. In the three patients, the offsets between the SVCs and IVCs were 0, 0.5, and 1 IVC diameters, respectively. The datasets for CFD simulations were collected before their discharge. The three-dimensional (3D) geometries of the Fontan pathway were reconstructed in Mimics 19.0 (Materialise, Haasrode, Belgium). Then, we used Mimics to draw the centerlines of these models. All TCPC vessels were cut proximal to the closest branching points. The selection rules of insert positions were the same as the idealized models, but owing to the specific 3D geometries, not all the insert positions were suitable for the future operations. Thus, we deleted some insert positions that were impossible to apply intraoperatively.

### Fluid domain meshing and computational fluid dynamic solver

All geometries were imported into ANSYS-ICEM 17.0 (ANSYS Inc., Canonsburg, PA, US) to generate unstructured meshes. All inlets and outlets were extruded over a sufficient distance to reduce the impact of the uncertainty of the boundary conditions on the region of interest and to increase the stability of the solver at the outflows^19^. A tetrahedral mesh was generated in the central connection area with a base mesh size of 0.6 mm and three boundary-fitted prism layers were generated at the near-wall regions to improve the resolution of the relevant scales in fluid motion. Mesh density function in the ANSYS-ICEM was used to increase the density of the grids. Each model grid was refined at the anastomosis between the conduit and vessel wall. The cell count for each model consisted of approximately 9 million cells.

CFD simulations were performed on Ansys-CFX 17.0. Convergence of simulations was based on the residual mean square (RMS) and the residual target was set as 1 × 10^−5^. The conservation target was set as 1 × 10^−4^. In idealized models, the flow rates of the venae cavae was 2.1 L/min, which are reasonable estimates of TCPC flow in children^20–22^. The static pressure at the inlets of the self-powered conduit was mean arterial pressure of patients, while outlets of the extruded LPA and RPA was set as 10 mmHg (1333 Pa). In patient-specific models, the boundary conditions of patients were from their clinical data. After induction of general anesthesia, mean SVC pressure, mean IVC pressure, and pulmonary arterial pressures were measured using a catheter inserted via the jugular vein into the pulmonary arteries. Time average flow rates of venae cavae were measured by cardiac MRI. The pressures at the SVC and IVC were measured by the average pressure of their cross sections. The cross sections locate at the closest branching points of each vessels. Pressure drop describes the pressure difference between two points. In current study, we used pressure drop to describe changes of the relative pressures between venae cavae and pulmonary arteries. Hepatic flow distribution (HFD) was quantified by examining the percentage of IVC flow entering the LPA and RPA in each patient-specific model.

## Results

### Idealized models

#### Pressure, pressure drop, and pulmonary flow distribution

**Figure 4:**
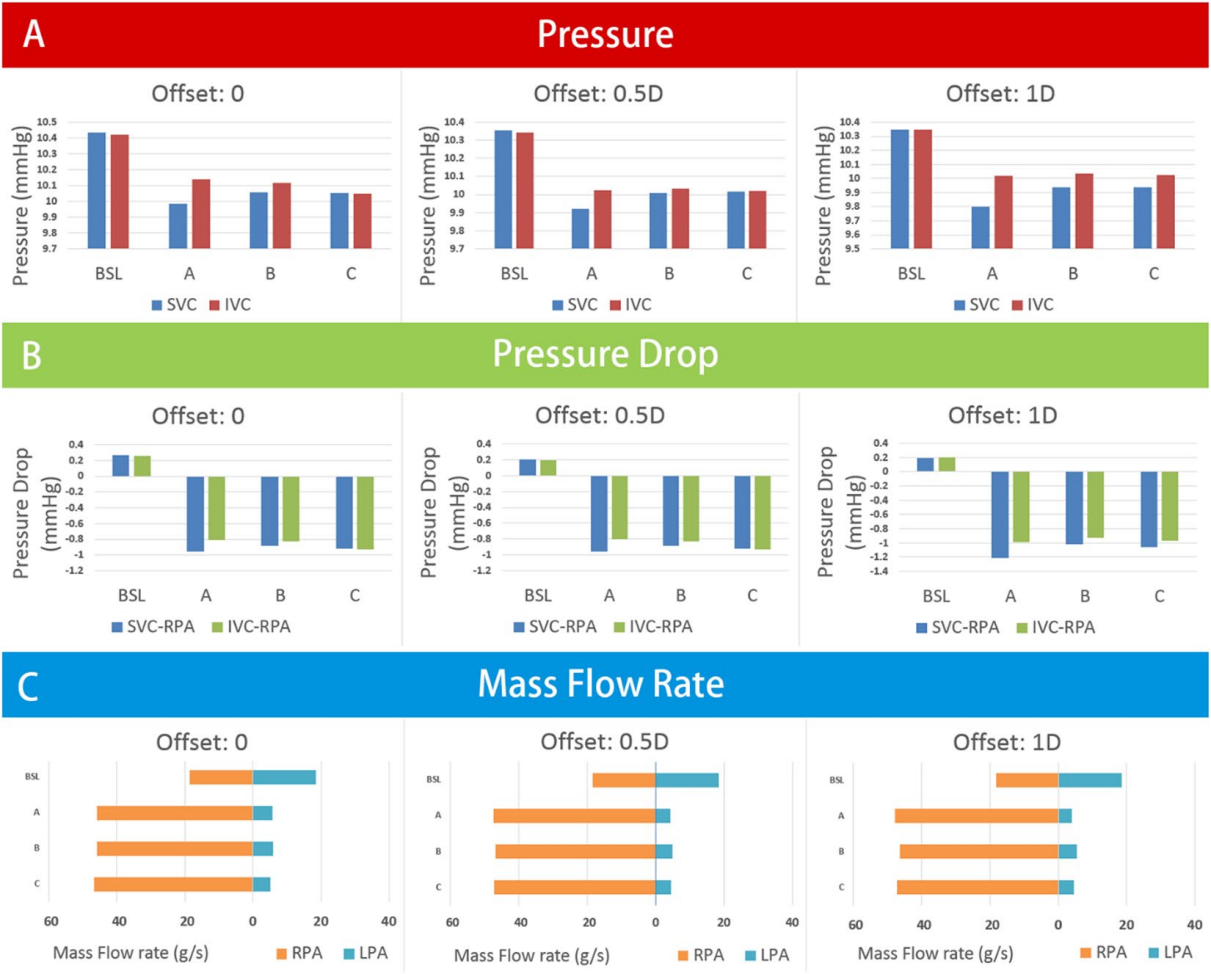
Comparing to baseline, the pressures of SVCs and IVCs were lower. Meanwhile, the pressure drops between venae cavae and pulmonary artery turned negative. **Section A** is the pressures at SVCs and IVCs. **Section B** is the pressure drops between venae cavae and RPAs. **Section C** is the mass flow rates at LPAs and RPAs. *BSL*, baseline; *SVC*, superior vane cava; *IVC*, inferior vena cava; *LPA*, left pulmonary artery; *RPA*, right pulmonary artery.

The pressures at SVCs and IVCs, the pressure drops between the venae cavae and RPAs, and pulmonary flow rates are shown in Fig. 4. The pressures at the SVCs and IVCs were lower than those at baseline. It was observed that all the conduits inserted from different points could drop the pressures at the SVCs and IVCs by approximately 0.3 mmHg. However, the degrees of reductions were different, and the optimal effect of decreasing the pressure at the SVCs was the conduit from point A. Moreover, the pressure drops between the venae cavae and pulmonary arteries became negative (changed from 0.2 mmHg (baseline) to −0.9 mmHg (experimental group)) after adding the conduits.

The pulmonary flow distribution data of the pulmonary arteries are shown in Fig. 4C. Because all the outlet directions of the conduits were pointed to the midpoints of RPA entrances, the mass flow rates of the RPAs were increased. Meanwhile, the Venturi effect also influenced the mass flow rates of the LPAs. Comparing to baseline models, the mass flow rate of LPAs in experimental group decreased to approximately 35%.

### Patient-specific models

#### The geometries of patient-specific models

**Figure 5:**
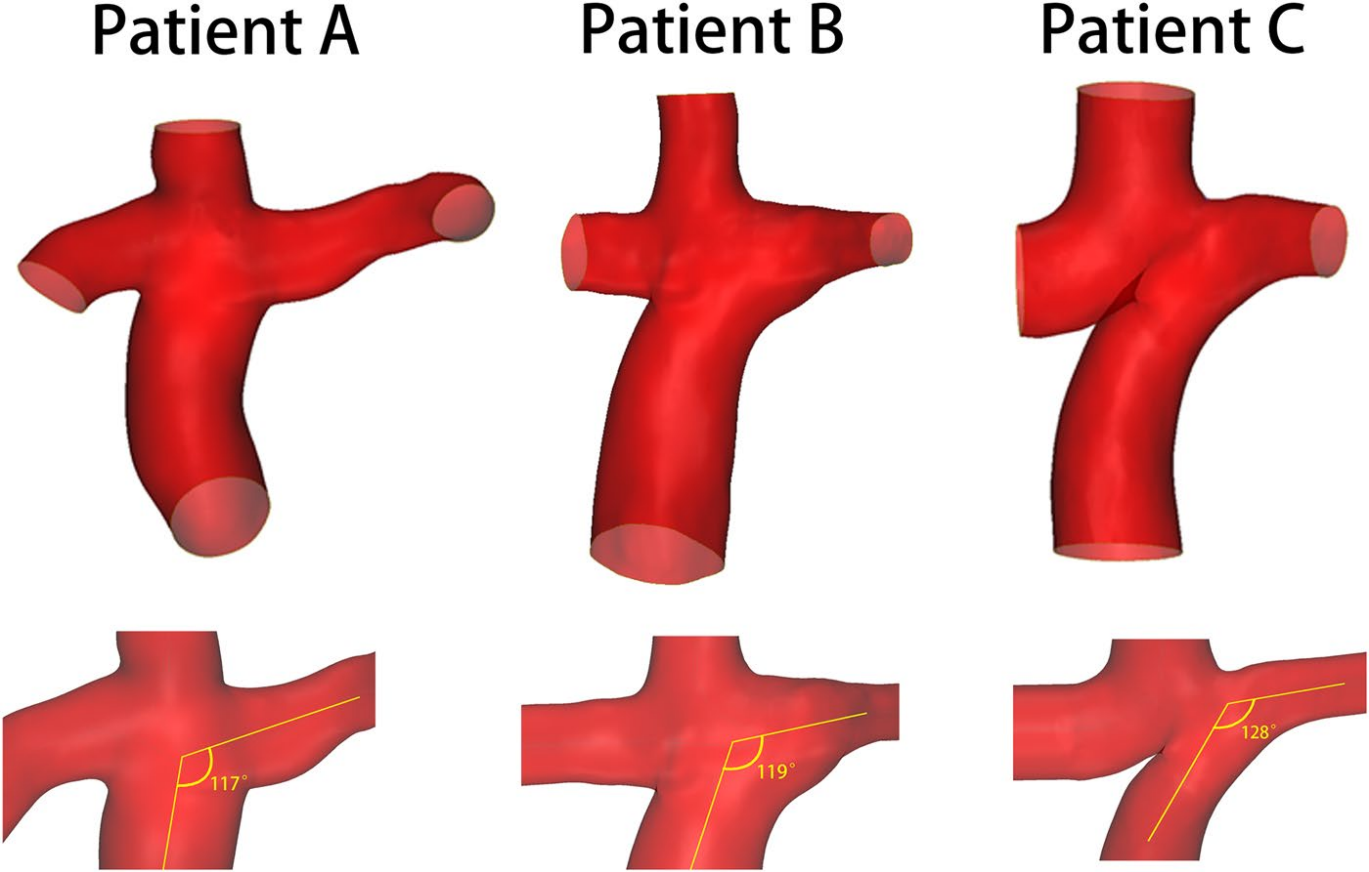
The full-range anatomic reconstruction and the angles between IVCs and LPAs of three extracardiac Fontan patients. The angles between IVCs and LPAs in patient-specific models. Patient A: 117°. Patient B: 119°. Patient C: 128°.

The geometries of the patient-specific models are shown in Fig. 5. All the angles between the IVCs and LPAs were obtuse 117°, 119°, and 128°, respectively.

#### Pressure, pressure drop, and pulmonary flow distribution

**Figure 6:**
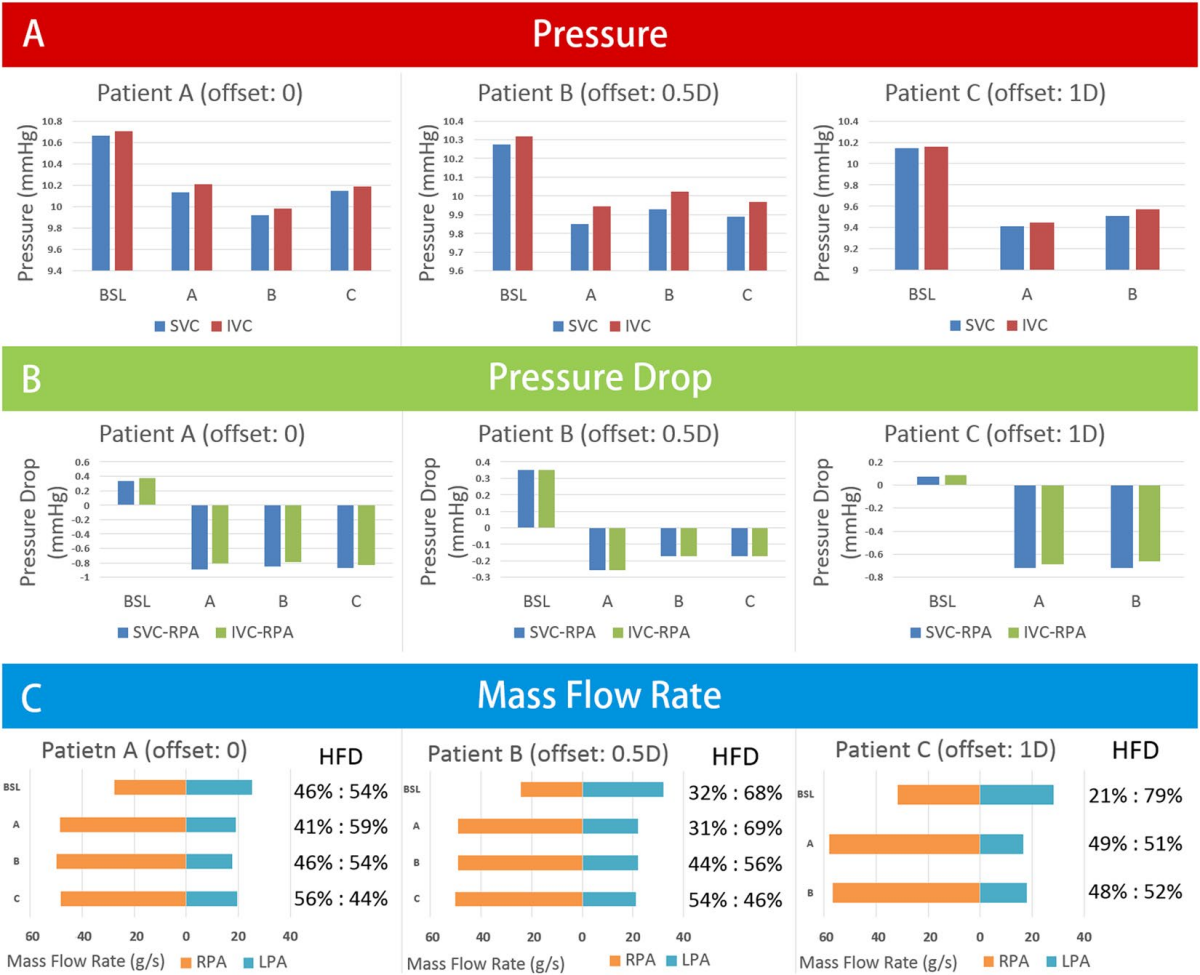
Section A is the pressures at SVCs and IVCs. Section B is the pressure drops between venae cavae and RPAs. Section C is the mass flow rates at LPAs and RPAs. *BSL*, baseline; *SVC*, superior vane cava; *IVC*, inferior vena cava; *LPA*, left pulmonary artery; *RPA*, right pulmonary artery; *HFD*, hepatic flow distribution.

**Figure 7:**
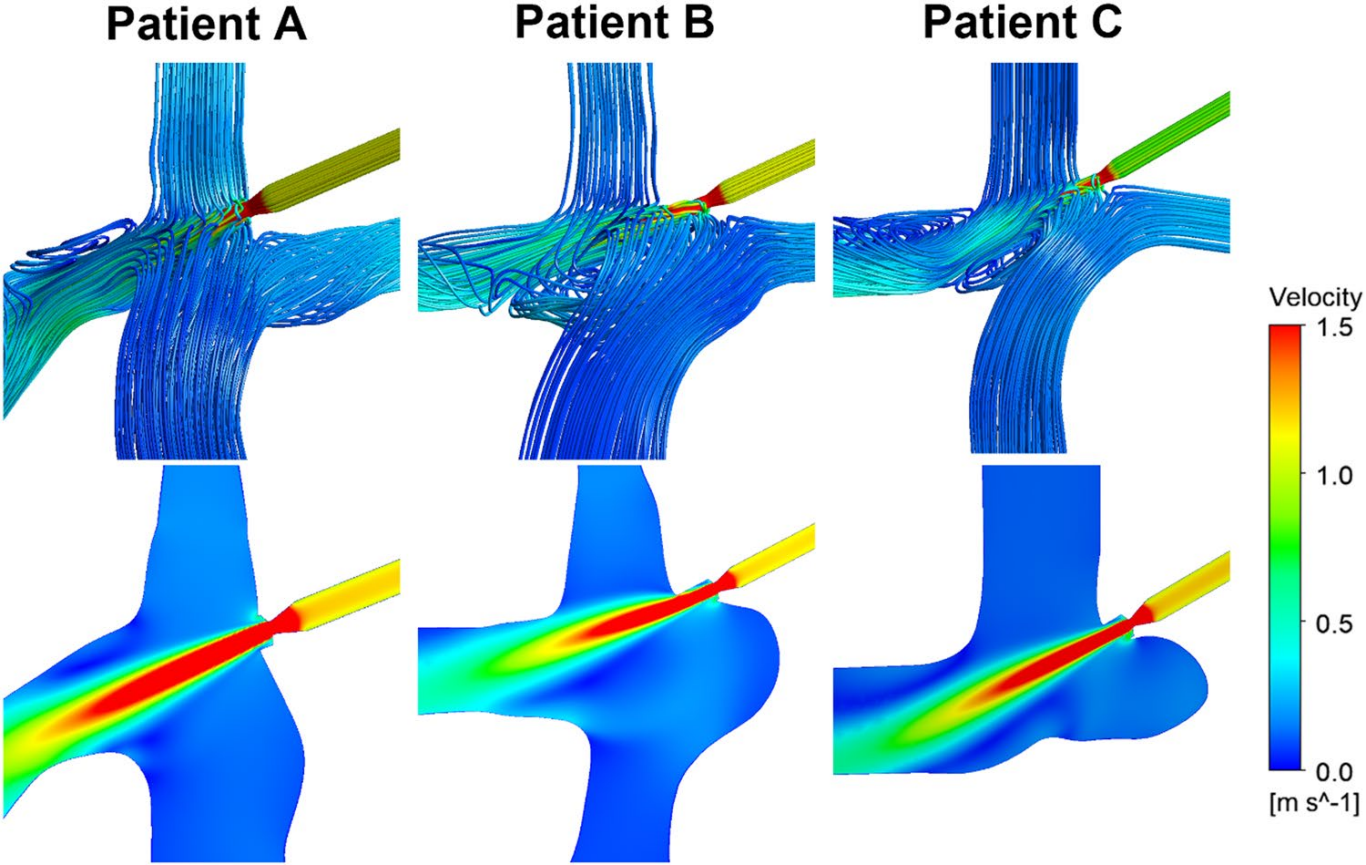
This figure shows the optimal results of three patients with the conduit.

Generally, in all patient-specific models, the conduits could drop approximately 0.5 mmHg at the venae cavae (Fig. 6A). The pressure drops between the venae cavae and RPAs in three patients were approximately −0.75 mmHg (Fig. 6B). The pulmonary flow distribution data of the RPAs increased by approximately 20 ml/s, while flow reduction of the LPAs was relatively limited (Fig. 6C). The jet from the conduit significantly changed the flow pattern around it (Fig. 7). The jet had a higher momentum compared to the surrounding blood, and the surrounding blood was carried along with the jet in this process.

In patient A, the conduits inserted from three points could decrease 0.49~0.74 mmHg. In the case of insertion point B, a good result is that the pressure of the vena cava is reduced by 0.7 mmHg, and the effect on the LPA blood flow is relatively limited. The HFD of patient A was slightly changed in self-powered models.

In patients B and C, the offsets of these two patients were 0.5 D and 1 D, respectively, which accounts for the majority of clinical Fontan patients. In these two patients, the effects of the conduits were consistent and the pressure drops between the SVCs and RPAs were approximately 0.7–0.85 mmHg. Moreover, the effects of the conduits on the blood flows to the LPAs were also limited, which were different from the idealized models. The baseline HFDs of patient B (LPA:RPA = 32%:68%) and patient C (LPA:RPA = 21%:79%) were imbalance, but after adding the self-powered conduit, HFD were balanced (patient B, LPA:RPA = 54%:46%; patient C, LPA:RPA = 49%:51%).

## Discussion

In this study, we designed a conduit to provide a self-powered circulation in extracardiac Fontan, which may help to alleviate the chronic elevation in systemic venous pressure following the Fontan procedure. We performed CFD analysis to evaluate different insert position of the shunt graft and calculate the fluid fields under physiological conditions to determine the optimal position. The features of this study include the following: (1) extracardiac Fontan with different offsets were included; (2) different insert positions were simulated; (3) the conduit could provide kinetic energy for the circulation, reducing the pressure of the venae cavae, which could increase the flow of pulmonary arteries without increasing pressures at the venae cavae; (4) both the spatial structures and feasibility of inserting a single conduit were taken into account, which makes the self-powered Fontan procedure applicable in clinical practice.

The decreased pressure in the venae cavae can vary from 0.3 mmHg to 0.8 mmHg according to different models. Given that the original pressure differences exist between the venae cavae and pulmonary arteries, the conduit can provide sufficient energy for the venous blood despite the limited reduction in pressure. Jet from the conduit significantly changed the flow pattern around it (Fig. 7). The jet had higher momentum compared to surrounding blood, and the surrounding blood was carried along with the jet in this process. It not only improved the kinetic energy for the blood but also formed a subpulmonary ventricle function, which meant that pressures at the venae cavae were lower than those of the pulmonary arteries.

An analysis of the results from idealized and patient-specific models shows that there are different influences on LPA flows by the conduits. In idealized models, resulting from the Venturi effect from the conduits, LPA flows were obviously decreased in some models. This is considered an unsatisfactory condition, because uneven distribution of blood from the IVC may cause pulmonary arteriovenous malformations^23,24^. However, in patient-specific models, there is relatively less influence on the LPA flows caused by the conduits, which may be due to patients’ individualized anatomical morphology. The angles of IVCs and LPAs in idealized models are 90°, whereas those in patient-specific models are larger than 90° (Fig. 5). In most of the Fontan patients, the angles were obtuse, because the artificial tunnels need to cling to the right atriums and then connect to the pulmonary arteries. Thus, blood from the IVC is likely to flow into the LPA, which is also associated with reduced energy loss in the TCPC^6^. Furthermore, there exists energy loss in the regions between the venae cavae, which may result in less blood from the IVC flowing into the RPA^6^; nevertheless, the conduit could generate an additional force to assist blood flow into the pulmonary circuit.

This inserted conduit could introduce pulsatile flow into pulmonary arteries, which helps to maintain the expression of endothelial nitric oxide synthase^25^. Moreover, such pulsatile blood flow has an advantage in maintaining endothelial function and preventing steady flow-induced pulmonary hypertension. Lack of pulsatile flow will result in elevation of pulmonary artery pressure, pulmonary vascular resistance^26,27^, and decreased endothelial-dependent vasorelaxation response of the pulmonary arteries^25^, which has a pernicious effect on Fontan circulation. Although the mass flow rate of RPA was significantly increased in this study, pressure of the venae cavae would not reach the criterion of pulmonary hypertension even if pulmonary vascular resistance was relatively high^16,17^.

While there are several publications quantifying outcomes use similar device to improve single ventricular circulation, these studies have had inappropriate insert positions^16^ or ignored the clinical feasibility^17^. Mahdi and colleagues used a Venturi jet to assist bidirectional Glenn in a previous study, but the increases in pulmonary flow were generally accompanied by increases in superior vena cava (SVC) pressure^16^. The conduit in that study was connected to SVC rather than the anastomosis of SVC and RPA, which result in increasing pressure of SVC. Ni and colleagues designed a self-powered Fontan circulation^17^. However, the nozzles were extended into pulmonary arteries without supporting structures, where displacement would occur. And they did not assess the influence of offsets and different insert positions of the conduit and did not apply the conduit to patient-specific models. Our study selected another connection area and assessed effects of different positions. Although our design is a shunt pointing to RPA, this structure is a clinically feasible structure and 4 mm diameter conduit is easy for anastomosing.

In this 3D model study, we mainly focused on hemodynamic phenomena in local vascular regions. However, 3D model cannot sufficiently account for the influence of the downstream vascular systems. Meanwhile, the hemodynamic performance of Fontan circulation is influenced by cardiovascular properties, such as pulmonary vascular resistance and left ventricular function^28^. But most clinical measurement cannot provide parameters of cardiac function and loading conditions. Liang and colleagues provide a method of patient-specific assessment to overcome these difficulties^29^. Therefore, patient-specific multiscale modeling of Fontan circulation could provide global hemodynamic profile. In our further study, Fontan patient-specific 3D models will be coupled with a reduced-dimensional model to address the interaction between local and global hemodynamics.

The most notable challenge for the application of the conduit is the prevention of thromboembolism. In this study, we considered blood as a homogeneous fluid, with fixed values for density and dynamic viscosity, and the destruction of blood cells was not considered. Disturbed flows occur in the regions of RPAs near the IVCs in both idealized models and patient-specific models, which may be caused by the rapid blood flow near these areas (Fig. 7). Furthermore, at the location where the conduit narrows, shear stress of the blood fluid significantly increased with velocity. Hellums and colleagues found that platelets experiencing a shear accumulation of > 3.5 Pa·s will undergo activation with subsequent release of prothrombotic messengers^30^. Therefore, thromboembolic events may be the greatest challenge for application of this pipeline *in vivo*. The use of aggressive anticoagulation measures and biocompatible materials could reduce the risk of thromboembolic events. To say the least, the circulation could still maintain a normal Fontan circulation irrespective of thrombosis occurring within the inserted conduit. Clinical application of this conduit-assisted Fontan requires further technical refinements and investigations (i.e. experimental study in animal models); however, it provides an alternative clinically feasible solution to help the subset of survivors overcome the unavoidable sequelae of Fontan circulation and have improved quality of life and longevity.

### Limitations

The small sample size in this study is one of the limitations; more patient-specific models are needed to verify the results. Another limitation is that steady inflow rates were imposed on the inlets, which means that the effects of respiration and exercise were not included. Moreover, outflow boundary conditions were imposed at the outlets in the CFD models, modeling the immediate perioperative status in the TCPC, and not the longer-term adaptation of the single ventricle physiology using resistances or lumped parameter networks in the boundary conditions. Finally, we assumed that the vessel walls were rigid and impermeable, with no-slip boundary conditions. A fluid-structure-interaction analysis is required in future studies.

## Conclusion

Using CFD simulation, we analyzed the effectiveness of a Fontan self-powered conduit. This conduit is a feasible, economical, and effective solution that could decrease vena cava pressures and increase the flow to the pulmonary arteries.
